# Modelling the Spatial Dependence of Multi‐Species Point Patterns

**DOI:** 10.1002/ece3.71066

**Published:** 2025-03-05

**Authors:** Chathuri L. Samarasekara, Ian Flint, Yan Wang

**Affiliations:** ^1^ School of Science RMIT University Melbourne Victoria Australia; ^2^ School of Agriculture, Food and Ecosystem Sciences The University of Melbourne Parkville Victoria Australia

**Keywords:** log‐Gaussian cox process, multivariate, pair correlation function, point process, saturated pairwise interaction Gibbs point process, semi‐parametric

## Abstract

The study of the spatial point patterns in ecology, such as the records of the observed locations of trees, shrubs, nests, burrows, or documented animal presence, relies on multivariate point process models. This study aims to compare the efficacy and applicability of two prominent multivariate point process models, the multivariate log Gaussian Cox process (MLGCP), and the saturated pairwise interaction Gibbs point process model (SPIGPP), highlighting their respective strengths and weaknesses when prior knowledge of the underlying mechanisms driving the patterns is lacking. Using synthetic and real datasets, we assessed both models based on their predictive accuracy of the empirical K function. Our analysis revealed that both MLGCP and SPIGPP effectively identify and capture mild to moderate clustering and regulations. MLGCP struggles to capture repulsive associations as they intensify. In contrast, SPIGPP can well estimate both the direction and magnitude of interactions even when the model is misspecified. Both models present unique advantages: MLGCP is particularly effective when there is a need to account for complex, unobserved heterogeneities that vary across space, while SPIGPP is suitable when interactions between points are the primary focus. The choice between these models should be guided by the specific needs of the research question and data characteristics.

## Graphical abstract

This study is the first to systematically compare the multivariate log‐Gaussian Cox process and the saturated pairwise interaction Gibbs point process, providing key insights into their application in ecological modelling. Our findings offer practical guidance for researchers, helping them select and apply these models effectively.

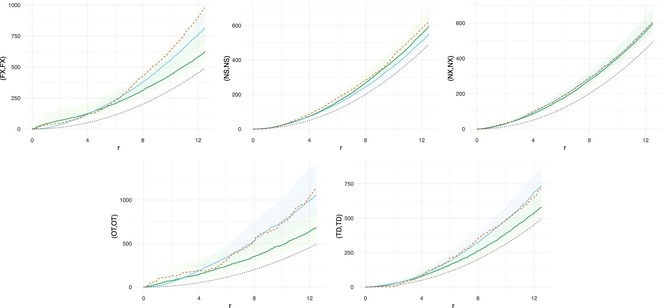

## 1 Introduction

Spatial point patterns are often studied in ecology to better understand the spatial arrangement of trees, animals, or other organisms. Point process models (PPMs) offer a theoretical framework for the understanding and analysis of what drives these patterns. In ecological studies, multivariate point process models are especially valuable because they can model multiple species simultaneously. Unlike single‐species models that assume species distributions are independent of one another, multivariate point process models allow for species interactions. This is particularly important in ecology, since species rarely exist in isolation.

For multi‐type point patterns, data points often exhibit spatial dependence, such as clustering or regularity. Clustering occurs when points are more likely to occur in close proximity to one another. Conversely, regularity arises when points are less likely to occur near each other. These spatial associations can be driven by shared, measurable predictors, such as elevation and soil, as well as unmeasured factors or intrinsic interactions between species. Accurately modeling spatial dependence not only enhances model performance but also provides valuable insights into the mechanisms underlying spatial patterns. This understanding is crucial for informed decision‐making in ecology.

While multivariate spatial point process models provide a richer understanding of spatial dependence, most multivariate spatial point process applications in ecology so far have been descriptive, relying on cross‐summary statistics such as cross K, cross pair correlation, or cross J functions (Baddeley et al. 2014b; Cronie and van Lieshout 2016; Møller and Waagepetersen 2003). Recent developments have introduced two main multi‐species point process models: the multivariate log‐Gaussian Cox process (MLGCP) (Waagepetersen et al. 2016; Choiruddin et al. 2020; Hessellund et al. 2022a) and the saturated pairwise interaction Gibbs point process (SPIGPP) (Rajala et al. 2018; Flint et al. 2022).

The main distinction between these two models is their mechanisms to model spatial dependence between points. Specifically, the MLGCP models' the clustering or regularity through latent random fields, which are driven by environmental or unobserved ecological factors. The SPIGPP, on the other hand, models spatial dependence between points through explicit functions that describe attractive or repulsive interactions between points directly. Throughout the paper, we use terms such as clustering and regularity to describe the observed spatial patterns in the data and attraction and repulsion to refer to the mechanisms or models that explain or generate these patterns. While the MLGCP and the SPIGPP model spatial dependence use different approaches, they are both valuable tools for studying multivariate point patterns. Both models have been used to study the well‐known Barro Colorado Island (BCI) (Waagepetersen et al. 2016; Rajala et al. 2018; Flint et al. 2022) and Washington DC crime data sets (Hessellund et al. 2022a).

While these models have seen increasing popularity in ecological community modelling, there has been a notable absence of direct comparative studies between the two types of point process models of MLGCP and SPIGPP. This may be due to their different theoretical foundations, which make direct comparisons challenging. The primary goal of our study is to evaluate and compare the ability of MLGCP and SPIGPP to account for and estimate the clustered or regular points in multi‐type point patterns, beyond what is attributable to measured predictors such as environmental or spatial covariates. For example, in a forest ecosystem, these models can help disentangle whether observed spatial clustering of species arises from shared responses to unmeasured environmental gradients or from direct biotic interactions, such as competition or mutualism. By simulating such scenarios, our study addresses how well these models can detect and represent this spatial dependence, guiding researchers in selecting the most appropriate model for their data. By comparing MLGCP and SPIGPP in these contexts, we aim to understand how each model captures the spatial dependencies in the data, whether driven by shared or unmeasured predictors, or by intrinsic interactions between points. This comparison provides valuable insights into the relative strengths and limitations of the models in representing complex spatial processes, offering guidance for their application in fields like ecology, epidemiology, and urban studies.

The paper is organised as follows: Section 2 includes an overview of multivariate log‐Gaussian Cox processes and saturated pairwise Gibbs processes and the detailed protocol for comparison of fitted models. In Sections 3 and 4, we applied the methodologies to the simulation studies and case analyses. Section 5 includes a detailed discussion of the results obtained from both the simulation study and the case study. Finally, Section 6 concludes with some closing remarks.

## 2 Materials and Methodology

In this section, we provide a brief overview of the MLGCP and SPIGPP models.

### 2.1 Multivariate Log Gaussian Cox Process

A log‐Gaussian Cox process (LGCP) is a Poisson point process with intensity given by $\Lambda \left(u\right)=\exp\left(\eta \left(u\right)+\kappa \left(u\right)\right)$, where $\eta \left(u\right)$ represents the fixed effects, often modelled as a linear function of covariates and $\kappa \left(u\right)$ is a zero‐mean Gaussian random field that capture the spatial dependence. In more detail, we recall the definition from Hessellund et al. (2022a), which builds upon the groundwork laid by Waagepetersen et al. (2016). We denote by $X=\left(X_{1},\ldots ,X_{p}\right),$ a multivariate spatial point process, where $X_{i}$ is a spatial point process on ${\mathbb{R}}^{d}$ (in ecology we will be using d=2) representing points of type i=1,…,p.

The point pattern $X_{i}$ for i=1,2,…,p is modelled as a Cox process with random intensity function:

(1) $$\Lambda_{i}\left(u\right)=\rho_{0}\left(u\right)\exp\left(\gamma_{i}^{T}z\left(u\right)+\sum_{k=1}^{q}\alpha_{\mathrm{ik}}Y_{k}\left(u\right)+\sigma_{i}U_{i}\left(u\right)\right)$$

The parameters are defined and interpreted as follows.

1. **Baseline intensity** $\rho_{0}$. It represents the underlying spatial trend, capturing large‐scale variations in the intensity unrelated to covariates or random effects.
2. **Environmental effect** $\gamma_{i}^{T}z\left(u\right)$. This term accounts for the effect of observed environmental covariates such as temperature or soil type, represented by $z\left(u\right)$, on the species' intensity. Here, $\gamma_{i}$ is the vector of coefficients that quantify how sensitive species i is to environmental factors.
3. **Latent factor effect** $\sum_{k=1}^{q}\alpha_{\mathrm{ik}}Y_{k}\left(u\right)$. This summation represents the effect of q unobserved latent Gaussian processes $Y_{k}\left(u\right)\left(k=1,\ldots ,q\right)$ that influence the intensities of all species types. Species are correlated due to their common dependence on the latent factors Y. Each $\alpha_{\mathrm{ik}}$ controls the influence each latent factor $Y_{k}$ has on the intensity of species i.
4. **Species‐specific variation** $\sigma_{i}U_{i}$. Here $\sigma_{i}U_{i}$ is a zero mean Gaussian random field with variance $\sigma_{i}^{2}$. It is used to capture species‐specific random variation (e.g., seed dispersal) that only affects the i th species.

The cross pair correlation function (pcf) of $X_{i}$ and $X_{j}$ is given by (Hessellund et al. 2022a);

(2) $$g_{\mathrm{ij}}\left(r;\theta \right)=\exp\left[\sum_{k=1}^{q}\alpha_{\mathrm{ik}}\alpha_{\mathrm{jk}}\exp\left(\frac{-r}{\xi_{k}}\right)+1\left[i=j\right]\sigma_{i}^{2}\exp\left(\frac{-r}{\psi_{i}}\right)\right]$$

where $1\left[i=j\right]$ is equal to 1 if i=j and 0 otherwise. θ is defined as the concatenation of $\alpha_{.k}={\left(\alpha_{1k},\ldots ,\alpha_{\mathrm{pk}}\right)}^{T}$ $\left(k=1,\ldots ,q\right)$, $\xi ={\left(\xi_{1},\ldots ,\xi_{q}\right)}^{T}$, $\sigma^{2}={\left(\sigma_{1}^{2},\ldots ,\sigma_{p}^{2}\right)}^{T}$, and $\psi ={\left(\psi_{1},\ldots ,\psi_{p}\right)}^{T}$. The g function is used to compare with the baseline value of 1 to detect clustering (>1) and regularity (<1) between types of points at distance r.

In Hessellund et al. (2022a), $\beta_{i}$, the coefficients of the covariates are estimated first using the first order conditional likelihood as used in Hessellund et al. (2022b). θ is then estimated by maximising the second‐order conditional composite likelihood function in Equation 7 in Hessellund et al. (2022a). This function, along with the pcfs in Equation (2), are invariant to some known transformations. However, the lack of identifiability is not a significant concern, given our focus on the resulting correlation structure rather than individual parameters $\alpha_{\mathrm{ij}}$'s. Further optimisation details can be found in Sections 3.1 and 3.2 of Hessellund et al. (2022a).

### 2.2 Saturated Pairwise Interaction Gibbs Point Process

The saturated pairwise interaction Gibbs point process (SPIGPP) models dependencies between points based on explicitly defined interaction functions. Points may either attract or repel each other, and the resulting spatial pattern emerges from the spatial interactions directly rather than due to latent environmental factors as in the MLGCP model. This section describes the SPIGPP introduced by Flint et al. (2022).

The model is specified by its density;

(3) $$f\left(X\right)\;=C\exp\left[\sum_{\left(x,i,m\right)\in X}\left(\beta_{0,i}+\sum \beta_{i,k}Z_{k}\left(x\right)\right)+\sum_{i=1}^{p}\sum_{z=\left(x_{1},i_{1},m_{1}\right)\in X}\alpha_{i_{1},i_{2}}u\left(z,{\left(X\backslash \left\{z\right\}\right)}_{i_{2}}\right)\right]$$

In the equation above, X is a spatial pattern, C>0 is a normalisation constant, and the set difference A\B is defined by {x: x in A and not in B}.

The other parameters are interpreted as follows.

1. **Environmental effect** $\beta_{0,i}+\sum \beta_{i,k}Z_{k}\left(x\right)$. This captures the impact of environmental factors, such as vegetation cover or temperature, represented by $Z_{k}\left(x\right)$, on the spatial pattern. The coefficients $\beta_{0,i}$ and $\beta_{k,i}$ quantify the strength of species i's response to environmental factors.
2. **Interaction effects** $\alpha_{i_{1},i_{2}}u\left(z,{\left(X\backslash \left\{z\right\}\right)}_{i_{2}}\right)$. This represents pairwise interactions between points, controlled by the interaction function $u\left(z,{\left(X\backslash \left\{z\right\}\right)}_{i_{2}}\right)$. Commonly used interaction functions are based on the Geyer, exponential decay, and bump interaction potentials, among others. More details can be found in Flint et al. (2022). The coefficient α determines the nature and strength of the intra‐ and inter‐species interactions. For example, if $\alpha_{i_{1},i_{2}}$ is negative, it indicates repulsion as seen in territorial species. Conversely, a positive $\alpha_{i_{1},i_{2}}$ could represent attraction, such as in plant species that form clusters.

Although SPIGPP appears complex, the model can be fitted using commonly used logistic regression methods by appropriately defining the dummy points. Detailed procedures for model fitting are provided in Flint et al. (2022).

### 2.3 Protocol/Algorithm for Comparison of Fitted PPMs

The primary objective of this study is to compare how well different point processes model clustering/regularity in data patterns when information about the underlying mechanism is lacking. However, due to the different nature of the MLGCP and SPIGPP models, direct comparison is not feasible. To allow for their comparison, we propose a step‐by‐step procedure.

In this study, we choose the K function as the basis for our comparisons because (1) it can accommodate various types of point patterns and can be easily adapted to different data types; (2) it effectively captures the degree of clustering or regularity at multiple scales; and (3) it is a non‐parametric statistic, meaning that it does not assume a specific distribution for the point process. This K function does not distinguish between different mechanisms underlying clustering or regularity; instead, it simply identifies evidence of these patterns based on the fitted model. While other functions like the J, L, and F functions, can also provide valuable insights, the K function's combination of interpretability, flexibility, and robustness makes it more suitable for our study. Additionally, it is straightforward to compare the fitted and empirical K functions against the baseline K function, representing the value of K for a homogeneous Poisson point process, defined as $K\left(r\right)=\pi r^{2}$. If the empirical K function is above (below) from the baseline K function, it indicates clustering (regularity) in the data.

To further enhance this analysis, we use simulation envelopes for the K‐function. These envelopes are constructed using Monte Carlo (MC) simulations of the fitted models, generating a range of expected values under the null hypothesis [The null hypothesis is that the observed point pattern is consistent with the fitted model, including its specified interaction structure (e.g., clustering or regularity)]. Comparing the observed K‐function with these simulation envelopes allows us to objectively evaluate how well each model replicates the observed spatial patterns. Deviations of the observed K‐function outside the simulation envelopes suggest discrepancies between the model and the data. This approach enables us to assess the ability of the models to reproduce clustering or regularity as evident in the data, providing a robust basis for comparison.

Therefore, we propose simulating N samples from the fitted model and subsequently computing MC estimates of the K function. This process can be easily implemented with the spatstat R package.

As a further step, we compute the Mean Integrated Squared Errors (MISE) aggregated over all cross‐type K functions, that is:
(4)

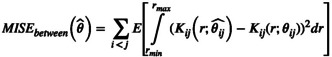

where for any pair of types i and j, the multitype K‐function $K_{\mathrm{ij}}\left(r;.\right)$ is the cross‐type K‐function (Baddeley et al. 2016). $r_{\min}$ and $r_{\max}$ define the range of distances r over which K function is evaluated. This definition can be extended to ${\text{MISE}}_{\text{within}}$ and ${\text{MISE}}_{\text{total}}$, which are similar to ${\text{MISE}}_{\text{between}}$ but with summation over i=j or i≤j.

## 3 Simulation Study

The objective of the simulation study was to evaluate and compare the effectiveness of two models—the MLGCP and the SPIGPP—in accounting for clustering and regularity in multi‐species point pattern data. This analysis was particularly focused on scenarios where the mechanisms driving spatial dependence were unknown or mis‐specified. We aimed to investigate how each model performed when the true data‐generating process differed from the assumed modeling framework. Specifically, we examined what happened when the data were generated from a MLGCP but modeled as a SPIGPP framework and vice versa. This comparison provided critical insights into the robustness and limitations of each model under mis‐specification.

In Appendix A, we revisited the five‐species simulation study presented in Waagepetersen et al. (2016) andHessellund et al. (2022a) beyond the bivariate case, focusing on evaluating the performance of the SPIGGP model when applied to data generated from the MLGCP model. To carry out this investigation, we used R (version 4.4.2) statistical software and the packages Multilogreg (version 0.1.0), randomFields (version 3.3.14), spatstat (version 3.0–6), ppjsdm (version 1.0), and ggplot2 (version 3.5.1).

### 3.1 MLGCP Scenarios

In each part of the simulation, we explored the association between two different species in various ways, focusing on both within and between species associations. Given that MLGCP cannot model regularity within a species, we designed four distinct scenarios, including mild to strong clustering patterns between and within species as well as mild to strong dispersed patterns between species. The scenarios were defined as follows:

1. MLGCP scenario 1—mild–moderate clustering between and within species (mild “+” b/w species).
2. MLGCP scenario 2—strong clustering between and within species (strong “+” b/w species).
3. MLGCP scenario 3—mild–moderate regularity between and mild to moderate clustering within species (mild “−” b & mild “+” w).
4. MLGCP scenario 4—strong regularity between and strong clustering within species (strong “−” b & strong “+” w).

As the initial step of our analysis, we simulated 100 MLGCP processes following the principles outlined by Hessellund et al. (2022a), using the parameters specified in Table A.1 in Appendix A. We then fitted these MLGCP scenarios using SPIGPP to evaluate its performance when applied to mis‐specified models.

**FIGURE 1:**
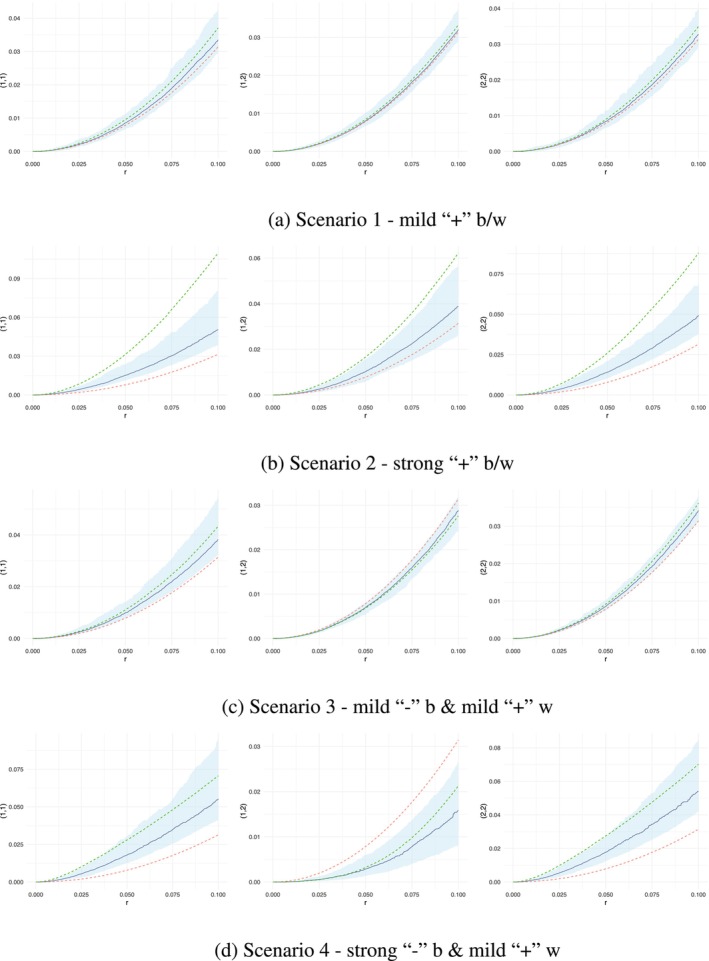
Comparison of K functions across simulated MLGCP scenarios fitted with SPIGPP models. Red dashed line—baseline K function ($\pi r^{2}$); green dashed line—empirical K function from the MLGCP data; blue line—point‐wise average of K function from SPIGPP fit; blue band—simulated envelope from the SPIGPP fit. Each row in the figure corresponds to a distinct scenario, labelled from 1 to 4, showcasing variations across different simulation setups. The y‐axis labels indicate the species being compared. For example, in the top‐left graph, the label (1,1) on the y‐axis represents the within‐species interactions of species 1. Similarly, the top‐middle graph, labelled (1,2), shows K functions between species 1 and species 2.

To assess the model fit, we compared the empirical K functions with the fitted K functions, along with their respective confidence bands. Although MLGCP and SPIGPP modelled clustering and regularity differently—through latent fields and interactions, respectively—the K function allowed us to compare the fits. Retrieving the model parameters for the MLGCP models was not emphasised, given the identifiability issues discussed by Hessellund et al. (2022a); Jalilian et al. (2020); and Choiruddin et al. (2020). Therefore, our primary focus was on the K functions when evaluating model performance. In Figure 1, we compared the fitted and empirical K functions against the baseline K function (shown as a dashed red line), which represented the value of K for a homogeneous Poisson point process, defined as $K\left(r\right)=\pi r^{2}$. If the empirical K function deviated above (below) this baseline K function, it indicated clustering(regularity) within/between species.

The K functions from scenario 1, featuring mild–moderate clustering between and within species, were depicted on the top row of Figure 1. The fitted SPIGPP model performed admirably in this scenario, with the fitted K functions (blue) closely aligning with all empirical MLGCP K functions (green) and falling well within the estimated confidence bands. We expected differences in the curve shapes of MLGCP and SPIGPP K functions, as they originated from two distinct underlying processes and were not anticipated to overlap.

The two bottom rows in Figure 1 showed MLGCP scenarios 3 and 4, respectively, involving between‐species regularity and within‐species clustering ranging from mild‐moderate to strong. SPIGPP adeptly captured the mild to moderate regularity between species (middle graph in the third row from the top of Figure 1) as well as the moderate attraction within the species (left and right graphs in the third row from the top). In MLGCP scenario 4 (bottom row of Figure 1), characterised by strong between‐species regularity and strong within‐species clustering, the SPIGPP model effectively captured the between‐species repulsive associations. It appropriately captured the strong within‐species attractions at longer distances, although at shorter distances, the SPIGPP fit slightly fell outside the confidence bounds.

MLGCP scenarios 2 (given in the second row from the top of Figure 1), characterised by strong clustering within and between species, present a unique challenge. Simulations from MLGCP under this scenario revealed significant variability in the number of points per species across samples. For example, some samples contained as few as 10 points for one species and over 100 for the other. This inherent volatility persisted even after removing potential outliers. When fitting SPIGPP models to these scenarios, this variability in the number of points affected the quality of the estimates, particularly underestimating the $\alpha_{p}$ values (i.e., interaction coefficients). Moreover, simulating from a fitted SPIGPP model with mild to large $\alpha_{p}$ values was particularly challenging. The Metropolis‐Hastings algorithm often failed to converge, leading to cases where one species goes extinct and did not reappear during the simulation. To mitigate these issues, we filtered out problematic samples, retaining 100 out of the initial 150 MLGCP samples for fitting SPIGPP models. Despite this pre‐processing, the number of points per species still exhibited substantial variation across retained samples, making reliable inference difficult.

### 3.2 SPIGPP Scenarios

Here, we once again considered two different species, focusing on both within species and between‐species associations. Since SPIGPP models could handle both attraction and regularity within species, we created five distinct scenarios in this section, covering mild to strong clustered and regular associations within and between species. The scenarios were defined as follows:

1. SPIGPP scenario 1—mild–moderate clustering between and within species (mild “+” b/w).
2. SPIGPP scenario 2—strong clustering between and within species (strong “+” b/w).
3. SPIGPP scenario 3—mild regularity between species and mild‐moderate clustering within species (mild “−” b & mild “+” w).
4. SPIGPP scenario 4—mild–moderate regularity between species and mild–moderate clustering within species $\left(2,2\right)$ and mild regularity within species $\left(1,1\right)$ (mild “‐” w/b & mild “+” w).
5. SPIGPP scenario 5—strong regularity between and strong clustering within species (strong “−” b & strong “+” w).

A detailed description of the simulation and fitting procedure was given in Appendix A. Consistent with the approach outlined in the previous section, we assessed the model performance of the MLGCP fit in mis‐specified scenarios by comparing the empirical and fitted K functions along with the respective confidence bands.

**FIGURE 2:**
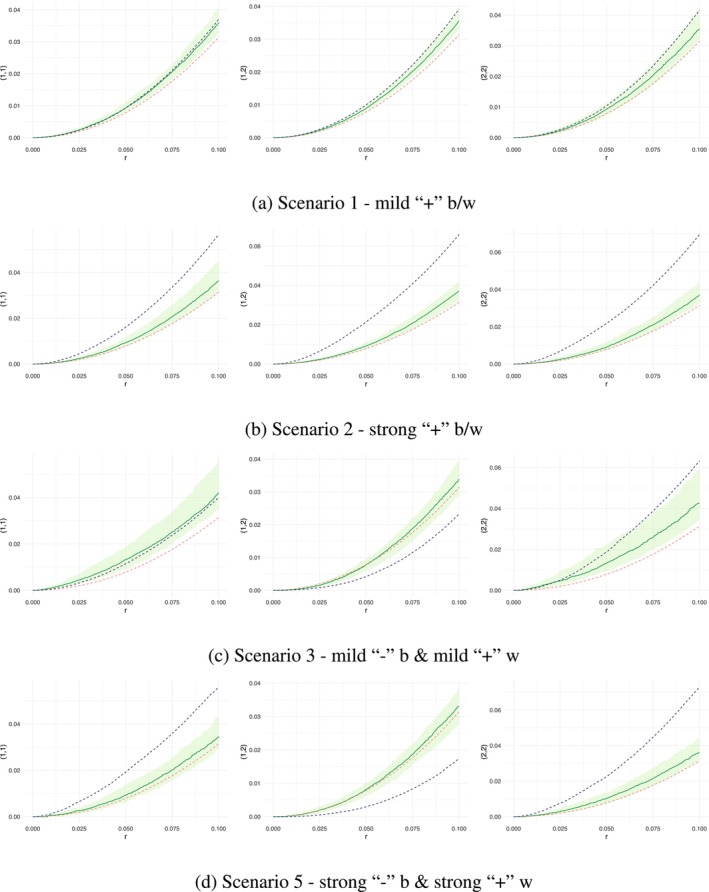
Comparison of K functions across simulated SPIGPP scenarios fitted with MLGCP models. Red dashed line—baseline K function ($\pi r^{2}$); blue dashed line—empirical K function from the SPIGPP data; green line—point‐wise average MLGCP K function; green band—simulated envelope from the MLGCP fit. Each row in the figure corresponds to a distinct scenario, labelled from 1 to 3 and 5, showcasing variations across different simulation setups. The y‐axis labels indicate the species being compared. For example, in the top‐left graph, the label (1,1) on the y‐axis represents the within‐species interactions of species 1. The top‐middle graph, labelled (1,2), shows the K functions between species 1 and species 2.

The top row in Figure 2 showed the comparison of K functions for the SPIGPP scenario 1, where there was mild–moderate clustering within and between the two species. The K functions on the top row of Figure 2 showed that the MLGCP model captured the mild to moderate within‐species attractions in the SPIGPP scenario well (the left and right graphs displayed the empirical K function in blue within the simulated envelope). However, the top middle graph, representing the between‐species interaction, showed the empirical K function at the upper bound of the envelope, indicating that the fit was not very accurate.

It was observed that the stronger the clustering generated by SPIGPP, the more challenging it became for the MLGCP fit to achieve the required magnitude of clustering both within and between species, even though it captured the presence of an attraction in the scenario (second row of Figure 2). This was similar to what we had observed in the previous section with MLGCP scenarios.

In the third row of Figure 2, we observed mild to moderate regularity between species and moderate clustering within each species (SPIGPP scenario 3). The MLGCP fit performed well when the clustering was mild, as seen in the left graph in the third row plots of Figure 2, and it also accurately estimated the clustering within the second species (right graph in third row) at short distances. While it identified the regularity between species at shorter distances, it was challenging for the MLGCP fit to accurately estimate the magnitude of the moderate regularity.

Similarly, in scenarios with strong regularity between species and strong clustering within each species (bottom row of Figure 2), such as Scenario 5, the MLGCP fit struggled to identify the regular pattern. It also found it challenging to accurately model the magnitude of the clustered as well as the regular associations in this scenario.

**FIGURE 3:**
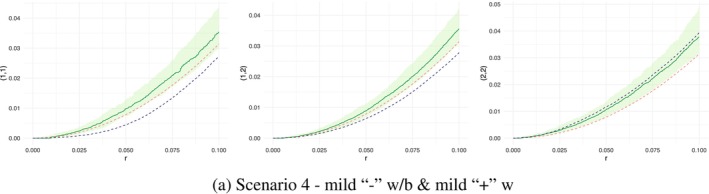
Comparison of K functions across simulated SPIGPP scenario 4 fitted with MLGCP models, where there is mild regulation between species and mild clustering and regulation within species. Red dashed line—baseline K function ($\pi r^{2}$); blue dashed line—empirical K function from the SPIGPP data; green line—point‐wise average MLGCP K function; green band—simulated envelope from the MLGCP fit. The y‐axis labels indicate the species being compared. For example, in the left graph, the label (1,1) on the y‐axis represents the within‐species interactions of species 1. Similarly, the middle graph, labelled (1,2), shows the K functions between species 1 and species 2.

In Figure 3, we observed the K functions generated for SPIGPP scenario 4, which featured a moderate clustering within species 2, mild regularity within species 1, and strong regularity between species $\left(1,2\right)$ (represented by the blue dashed line). The right plot in Figure 3 indicated a good fit for species 2, as the blue and green solid lines closely aligned and were within the envelope. However, this accuracy was not observed in the other two K functions (left and middle plots in Figure 3), where the regularity between the two species and within species 1 were inaccurately modelled as clustering by the MLGCP model. Although the MLGCP model was expected to struggle with capturing negative associations within species, it should, in theory, have been able to identify between‐species regularity. However, this was not observed in this scenario.

## 4 Case Study

In this section, we revisited the South Carolina Savannah river site study conducted in Flint et al. (2022). Studying the spatial patterns of plants was of significant interest to ecologists as it provided a better understanding of the community structure.

**FIGURE 4:**
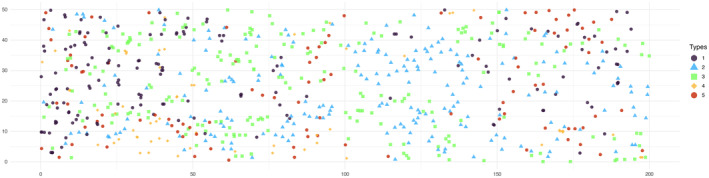
Trees in the Savannah River, South Carolina, USA. Abbreviations for the tree species are used as follows: FX, Carolina ash ( *Fraxinus caroliniana* ); NS, Swamp tupelo ( *Nyssa sylvatica* ); NX, Water tupelo ( *Nyssa aquatica* ); OT, stems of eight additional species; TD, Bald cypress ( *Taxodium distichum* ).

Seven different plots of the South Carolina Savannah river site were originally created by Bill Good (Good and Whipple 1982), and several analyses have been conducted thereafter (Good and Whipple 1982; Jones et al. 1994; Dixon 2002; Flint et al. 2022). In this study, we considered one of the plots from the original experiment (Figure 4). The dataset could be obtained using the R language (R Core Team 2024) as ecespa::swamp from the ecespa package available on CRAN.

The dataset, as shown in Table 1, contained four species of trees and another group of eight additional tree species (OT), with their arrangement shown in Figure 4. There were no known environmental covariates related to this dataset; however, the (unmeasured) water level was thought to be important for the spatial distribution. Therefore, we introduced an artificial horizontal covariate (equal to x/100, where x is the point's *x*‐coordinate) that was thought to be proportional to the water level (Flint et al. 2022). The fitting procedure used in the analysis is explained in detail in Appendix C.

**TABLE 1:** Trees in a plot in the Savannah River, South Carolina, USA.

Trees	Number
FX—Carolina ash ( *Fraxinus caroliniana* )	156
NS—Swamp tupelo ( *Nyssa sylvatica* )	205
NX—Water tupelo ( *Nyssa aquatica* )	215
OT—stems of 8 additional species	60
TD—Bald cypress ( *Taxodium distichum* )	98

The parameters ϕ and σ determine the volatility of the Gaussian random fields in the MLGCP model (Table C.2 in Appendix C). The estimates of $\phi_{i}$ for tree species were generally small, with Carolina Ash having the smallest value and Bald Cypress the largest. The estimates for $\sigma_{i}$ vary from small to large depending on the tree species. For instance, Carolina Ash and the ‘other tree’ category exhibit significant clustering, while Swamp Tupelo showed the least clustering. All other tree species displayed moderate levels of clustering.

**FIGURE 5:**
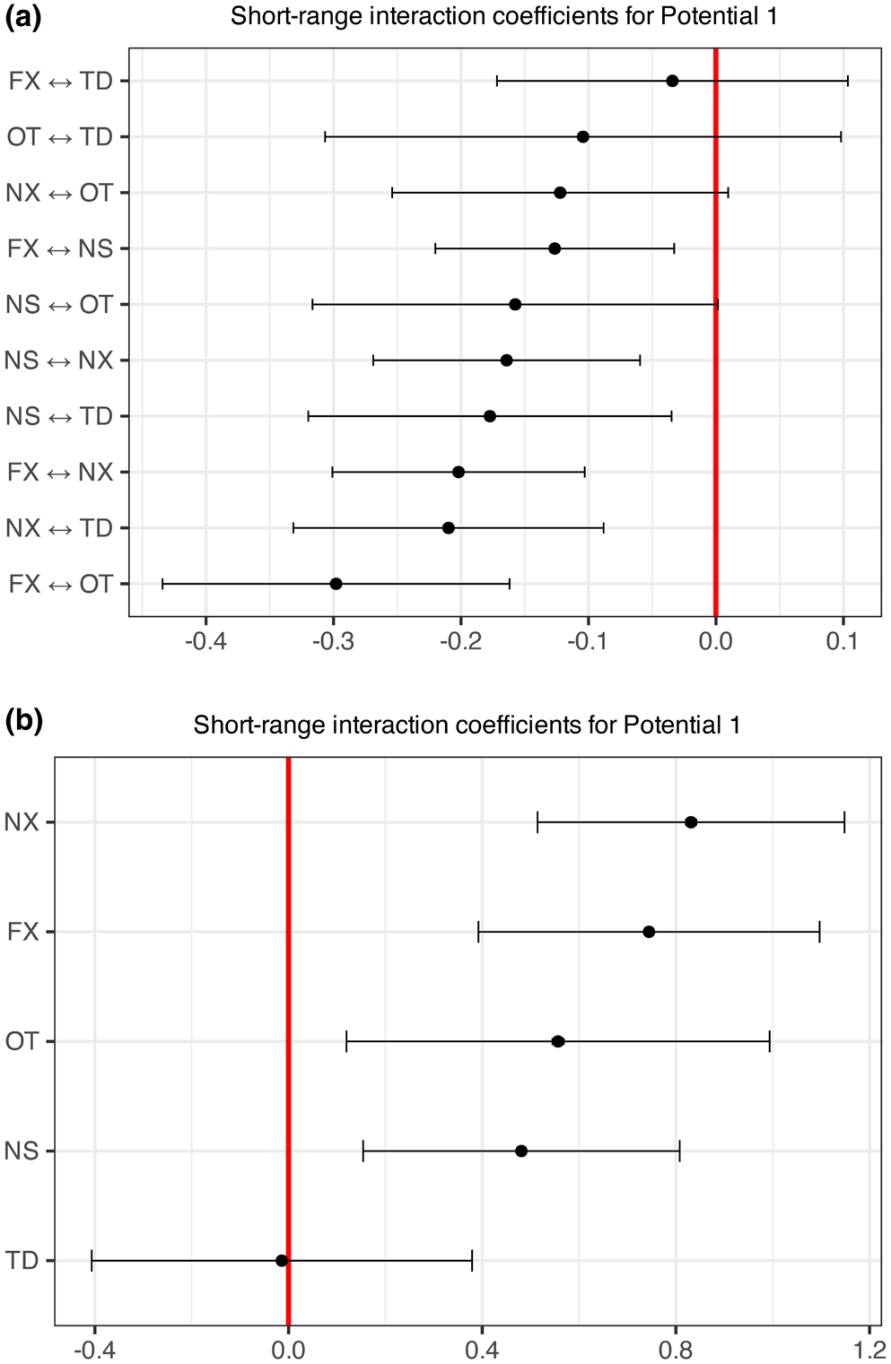
Estimated short range interaction coefficients for the tree types of SPIGPP fitted model. The coefficients of the within short‐range interactions ($\alpha_{p}$) of TD and the coefficients of the between short‐range interactions ($\alpha_{p}$) of $\left(\mathrm{FX},\mathrm{TD}\right)$, $\left(\mathrm{OT},\mathrm{TD}\right)$ are not statistically significant at 0.05 level of significance.

The coefficients and their significance for estimated short‐range interactions in the SPIGPP were presented in Figure 5. Notably, most of the coefficients of the short‐range interactions $\left(\alpha_{p}\right)$ were found to be statistically significant at 0.05 level of significance. Interaction coefficients for within species were given at the bottom, while the top graph showed the between species interaction coefficients. The within‐species interactions of Bald Cypress, along with the between‐species interactions of Bald Cypress and Other tree species, Water Tupelo and other species as well as Carolina Ash and Bald Cypress, were found to be statistically insignificant.

The within‐species short‐range interaction coefficients, except for Bald Cypress, were all positive and notably higher than the coefficients for between‐species interactions, highlighting stronger clustering associations within species. In contrast, all between‐species interactions exhibited negative coefficients, indicating regularity between different tree species. Among these, the weakest regularity was observed between Carolina Ash and Bald Cypress (−0.038), while the strongest regularity occurred between Carolina Ash and other tree species (−0.301). These patterns suggested a clear ecological preference for trees of the same species to cluster together, while interactions between different species were less favorable. This aligned with findings reported in the study by Flint et al. (2022).

**FIGURE C.1:**
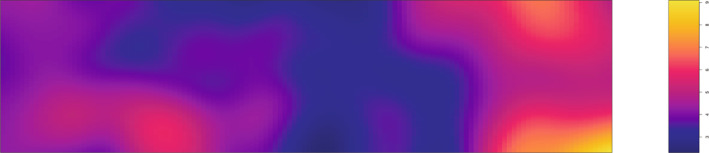
Estimated background intensity ($\rho_{0}$)—fitted MLGCP model.

Table 2 presents the estimated coefficients for the SPIGPP model, highlighting the significance of background intensity and water level effects for different tree species. The background intensity effect was significant and positive for most species (See Figure C.1) as expected. Additionally, the water level had a significant negative effect, suggesting that higher water levels may limit tree occurrence.

**TABLE 2:** Significance of covariates in the SPIGPP model. The standard errors of the estimates are given in brackets. The *'s indicate the significance codes, where 0 “***”, 0.01 “**”, 0.05 “*”.

	Intercept	Water level	Background intensity
FX	−4.65 (0.44)***	−0.88 (0.12)***	0.20 (0.09)*
NS	−3.92 (0.37)***	−0.21 (0.15)	0.03 (0.07)
NX	−4.99 (0.39)***	−0.43 (0.14)**	0.22 (0.07)**
OT	−5.67 (0.52)***	−0.90 (0.26)***	0.42 (0.11)***
TD	−5.46 (0.53)***	−0.58 (0.20)**	0.51 (0.12)***

The estimates derived from the MLGCP model using $\left(q,\lambda \right)=\left(2,2.5\right)$ were summarised in Table C.2 in Appendix C. The correlation scale parameter estimates for the common latent fields, denoted as ξ, were reported as $\left(1.44,21.05\right)$. Lasso regularisation drove the estimates of the $Y_{1}$ latent field, $\hat{\alpha_{.1}}$, to 0, similar to the results derived in Hessellund et al. (2022a), while the latent field $Y_{2}$ exhibited fluctuations in $\hat{\alpha_{.2}}$ from moderate to large. Swamp Tupelo and Water Tupelo responded negatively to $Y_{2}$, and they were negatively correlated with Carolina Ash, Bald Cypress, and other tree species.

**FIGURE C.2:**
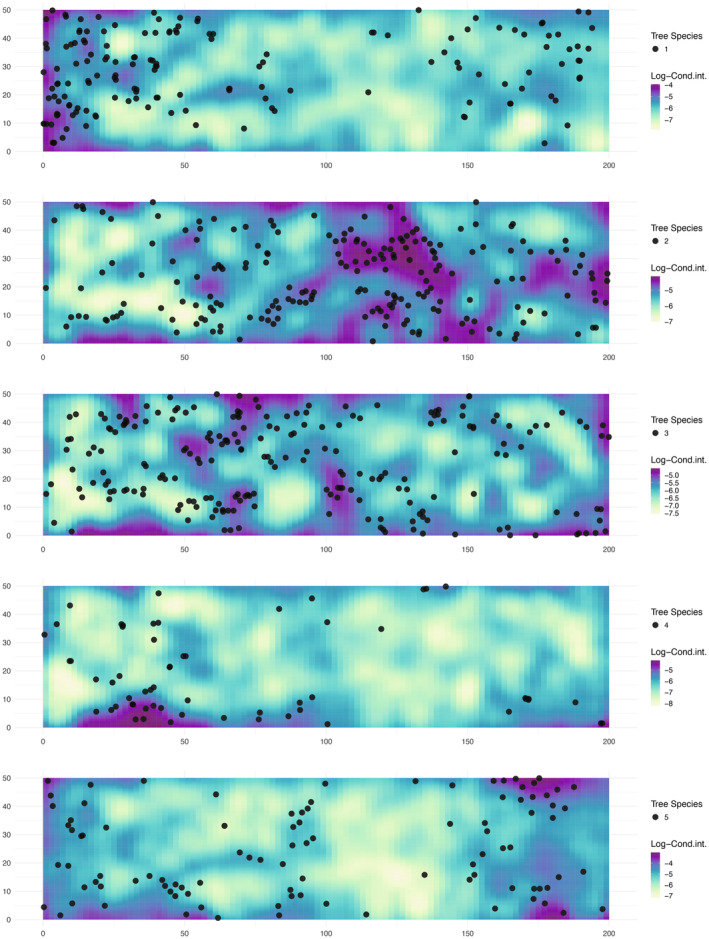
SPIGPP fitted model—The log‐Papangelou conditional intensities of each species, conditioned on all other species. The model effectively captures the spatial inhomogeneity, delineating regions of high and low individual density. This separation is well reflected in the AUC metric, which is notably high for these species: [Carolina Ash (0.703), Swamp Tupelo (0.605), Water Tupelo (0.609), other tree species (0.728), and Bald Cypress (0.679)].

The log‐conditional intensities (Baddeley et al. 2016; Daley and Vere‐Jones 2003) of a given species in the SPIGPP model, conditional on all other species, were given in Figure C.2 in Appendix C. The SPIGPP model effectively captured the spatial inhomogeneity, with its conditional intensity appropriately delineating the area into regions of high and low tree density. The rather large corresponding AUC values in SPIGPP for these species [Carolina Ash (0.703), Swamp Tupelo (0.605), Water Tupelo (0.609), other tree species (0.728), and Bald Cypress (0.679)] corroborated this result.

**FIGURE 6:**
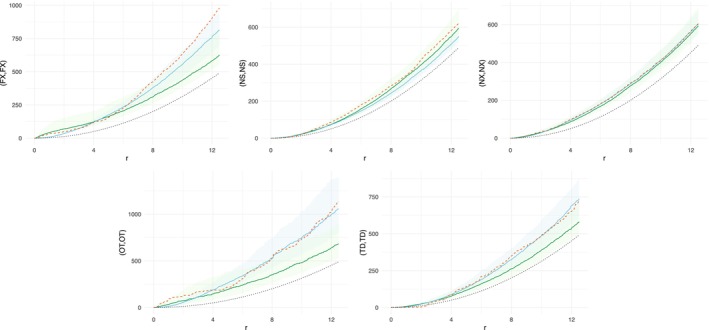
Comparison of empirical and fitted K functions for the swamp data. Black dotted line—baseline K function $\left(\pi r^{2}\right)$; dashed orange line—empirical K function from the data; green line—point‐wise average K function from the MLGCP model; blue line—point‐wise average K function from the SPIGPP model; blue band—simulated envelope from the SPIGPP model; green band—simulated envelope from the MLGCP model. The y‐axis labels indicate the species being compared. For example, in the top‐left graph, the label (FX, FX) on the y‐axis represents the within‐species interactions of species FX.

**FIGURE 7:**
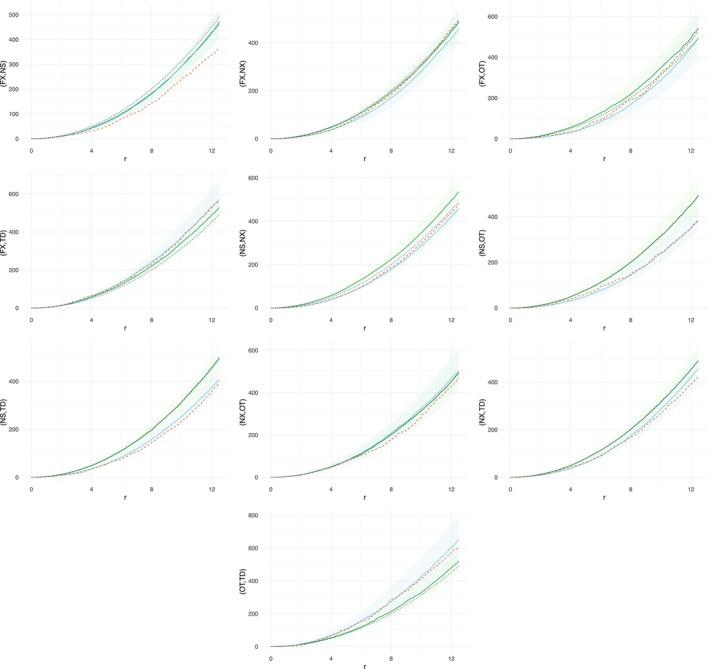
Comparison of empirical and fitted K functions for the swamp data. Black dotted line—baseline K function $\left(\pi r^{2}\right)$; dashed orange line—empirical K function from the data; green line—point‐wise average K function from the MLGCP model; blue line—point‐wise average K function from the SPIGPP model; blue band—simulated envelope from the SPIGPP model; green band—simulated envelope from the MLGCP model. The y‐axis labels indicate the species being compared. For example, in the top‐left graph, the label (FX, NS) on the y‐axis represents the within‐species interactions of species FX and NS.

Figures 6 and 7 displayed the respective K functions for the fitted models: MLGCP (green) and SPIGPP (blue). We computed the envelopes of the *K* function based on simulations from the fitted models of MLGGP and SPIGPP, which were shown in light green and light blue, respectively. Additionally, the empirical (dashed orange) and base K (dotted black) functions were shown for comparison.

In Figure 6, we displayed the within‐species associations of the five tree species, all of which showed positive associations. For Carolina Ash (top left) and other tree species (bottom left), both SPIGPP and MLGCP fit the data well at shorter distances (< 4 m). However, at longer distances (4 m–12 m), SPIGPP continued to capture species interactions effectively, while MLGCP failed to do so. For Bald Cypress (bottom right), the SPIGPP model provided a better fit compared to the MLGCP model. For Bald Cypress (bottom right), the empirical (dashed orange) K function was zero up until 2 m, as trees closer than 2 m to each other had been cut down by people at the time of measurement. Unfortunately, none of the models were able to accurately capture this change in the K functions. However, the SPIGPP was able to well capture the intra‐species interaction beyond distance of 2 m. For Swamp Tupelo (top middle), the MLGCP model showed a slightly better fit. Both MLGCP and SPIGPP models performed exceptionally well at modelling Water Tupelo (top right).

In Figure 7, the between‐species clustering/regularity is presented. We observed that the SPIGPP model provided a better fit than the MLGCP model for most of the between‐tree spatial associations shown in Figure 7. Most of the regular associations (top middle, top right graphs, third row graphs, second row middle and right graphs) were either estimated as clustering by the MLGCP model or were not accurately identified, defaulting to the baseline K function, while SPIGPP accurately captured these associations. In the top‐left graph showing the association between Carolina Ash and Swamp Tupelo, both the MLGCP and SPIGPP models failed to accurately capture the extent of the regularity. In the case of the attractions between Carolina Ash and Bald Cypress (second row, left) and other tree species and Bald Cypress (bottom row), the MLGCP model did identify the presence of clustering but struggled to estimate its magnitude, while the SPIGPP model provided precise estimates for both the associations and their magnitudes.

As shown in Table 3, the MISEs for SPIGPP were much smaller for both within and between species interactions. SPIGPP performed much better at modeling both between and within tree species associations. While the MLGCP did a fairly good job of modeling the within species associations, it was not as effective as SPIGPP. In summary, the SPIGPP model more accurately captured the spatial interactions and dependencies present in the data, leading to more reliable and interpretable results. This improved fit was evident across various distances, highlighting the effectiveness of the Gibbs process in modeling spatial point patterns, particularly in scenarios where clustering or regularity was present.

**TABLE 3:** MISE of fitted SPIPP and MLGCP models for the Savannah trees.

	SPIPP	MLGCP	Base
${\text{MISE}}_{\text{total}}$	715.76	3861.69	13,012.67
${\text{MISE}}_{\text{within}}$	1448.38	8841.47	43,036.64
${\text{MISE}}_{\text{between}}$	316.15	1145.44	1003.09

## 5 Discussion

In this study, we evaluated the strengths and limitations of the multivariate log‐Gaussian Cox processes and the multivariate saturated pairwise interaction Gibbs processes in capturing spatial dependence in multi‐type spatial data. By simulating data from one model and fitting it with the other, we explored how well each model could represent the observed spatial patterns under mis‐specification scenarios. Using metrics such as the cross K‐functions and mean integrated squared error (MISE), we assessed the quality of the model fits and their ability to capture the clustering and regularity featured in the data. These findings provide insights into the effectiveness and suitability of each model framework to analyze complex multi‐type spatial datasets, particularly when the underlying mechanisms driving spatial dependence are not fully known.

The MLGCP and SPIGPP are both robust frameworks for analyzing multi‐type point patterns, yet they model spatial dependence through fundamentally different mechanisms. The SPIGPP models spatial dependence directly through interaction functions acting on pairs of points, thereby highlighting patterns of clustering and regularity without requiring additional hypotheses on its underlying source. SPIGPP models are ideal for modeling regular spatial patterns, such as those subject to competition for space or self‐thinning. They can also model clustered spatial patterns, such as those resulting from seed dispersal around mother trees. In contrast, MLGCP models attempt to explain spatial dependence as emerging from environmental heterogeneity (observed or unobserved) and shine when modeling large‐scale clustering.

In our study, we first studied two‐species point patterns under different scenarios using mis‐specified models. Then, we expanded our analysis to a five‐species simulation study where we fitted the data generated by the MLGCP model using the SPIGPP model. Finally, we explored the South Carolina Savannah river swamp data. Through our studies, we found that both the MLGCP and the SPIGPP effectively identify and capture mild to moderate clustering and regularity in multi‐type spatial data. However, as the observed regularity becomes stronger, the MLGCP struggles to accurately represent these patterns. In contrast, the SPIGPP demonstrates the ability to estimate both the direction and magnitude of the associations between points, even when the data are simulated from an MLGCP model. A summary of the findings can be found in Table 4. Nonetheless, the SPIGPP faces challenges in modeling complex spatial associations, for example, those that shift from clustering to regularity. These challenges may be addressed by developing and fitting more complex SPIGPP models, such as including medium and/or long‐range interaction terms.

**TABLE 4:** Summary of comparative simulation study.

Scenarios	Fit with MLGCP		Fit with SPIGPP	
	Within	Between	Within	Between
Mild “+” b/w	Good	Good	Good	Good
Strong “+” b/w	Poor	Poor	Poor	Poor
Mild “−” b/w & mild “+” w	Good (clustering)	Poor	Good	Good
	—	—	Good (regularity)	—
Mild “−” b & mild “+” w	Good	Good	Good	Good
Strong “−” b & strong”+” w	Poor	Poor	Good	Good

When deciding between SPIGPP and MLGCP, we should also take the following into consideration.

- Gibbs processes often offer more direct interpretability regarding interaction parameters. In contrast, the MLGCP, while flexible and capable of capturing more complex patterns, can sometimes be less directly interpretable due to the latent Gaussian fields.
- The SPIGPP is highly effective in handling many species and points, accommodating approximately hundreds of species and up to ∼100,000 points. The MLGCP involves modelling latent Gaussian fields, which require operations on large covariance matrices. For high‐dimensional spatial domains, this becomes computationally expensive.
- The MLGCP can become over‐parameterised when modelling many species with shared as well as species‐specific random effects. This can lead to challenges in parameter estimation. Instead, the likelihood function for the SPIGPP model can be approximated by a pseudo‐likelihood, with the parameters estimated using standard logistic regression techniques (Baddeley et al. 2014a). This has greatly simplified computations for a spatial Gibbs process.
- It is also worthwhile to remember that Rajala et al. (2018) said “For longer spatial scales, the log‐Gaussian Cox process is a well‐suited modelling framework, but it is not a good framework for studying small‐scale interactions. Instead, we shall use the multivariate Gibbs point process model to discover small scale point‐to‐point interactions.”. This implies that the MLGCP excels in understanding patterns over larger areas, driven by latent spatial structures. Conversely, if the focus is on capturing local interactions between points, the SPIGPP would be the preferable option due to its ability to model explicit point‐to‐point dependencies.

## 6 Conclusions

This paper demonstrates that both the MLGCP and SPIGPP models can effectively be applied to multi‐species spatial patterns exhibiting various types of spatial dependencies. Each model performs comparably well with mild to moderate associations, accurately identifying and capturing these interactions. Notably, the SPIGPP models excel at identifying and capturing regular associations, while the MLGCP models are better at capturing strong clustered associations. The SPIGPP reliably identifies the direction of the associations, even if it struggles with the magnitude, whereas MLGCP models have a limited ability to identify regular associations, sometimes misinterpreting them as clustered associations.

## Author Contributions

**Chathuri L. Samarasekara:** conceptualization (supporting), data curation (lead), formal analysis (lead), methodology (lead), resources (supporting), visualization (lead), writing – original draft (lead), writing – review and editing (equal). **Ian Flint:** conceptualization (lead), data curation (supporting), methodology (lead), resources (lead), supervision (lead), visualization (supporting), writing – review and editing (equal). **Yan Wang:** conceptualization (lead), data curation (supporting), funding acquisition (lead), methodology (lead), resources (lead), supervision (lead), writing – review and editing (equal).

## Conflicts of Interest

The authors declare no conflicts of interest.

## Figures and Tables

**FIGURE B.1:**
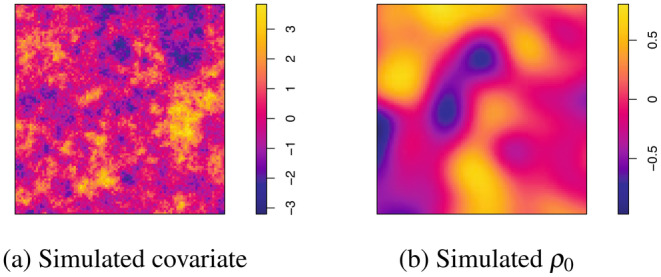
Realisations of covariate and $\rho_{0}$ for the simulated five‐variant MLGCP.

**FIGURE B.2:**
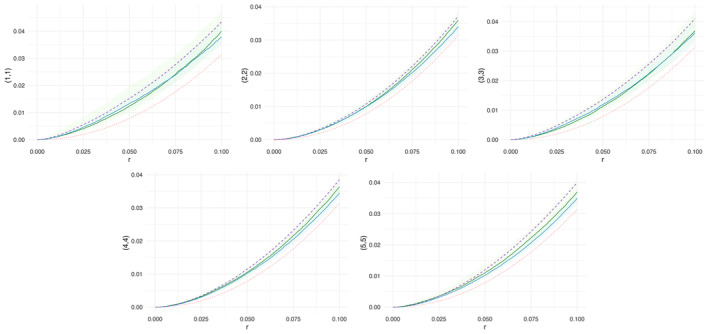
Comparison of empirical and fitted K functions for the five species simulated data. Red dotted line—baseline K function ($\pi r^{2}$); dashed purple line—empirical K function from the data; green line—point‐wise average K function from the MLGCP model; blue line—point‐wise average K function from the SPIGPP model; blue band—simulated envelope from the SPIGPP model; green band—simulated envelope from the MLGCP model. The y‐axis labels indicate the species being compared. For example, in the top‐left graph, the label (1,1) on the y‐axis represents the within‐species interactions of species 1.

**FIGURE B.3:**
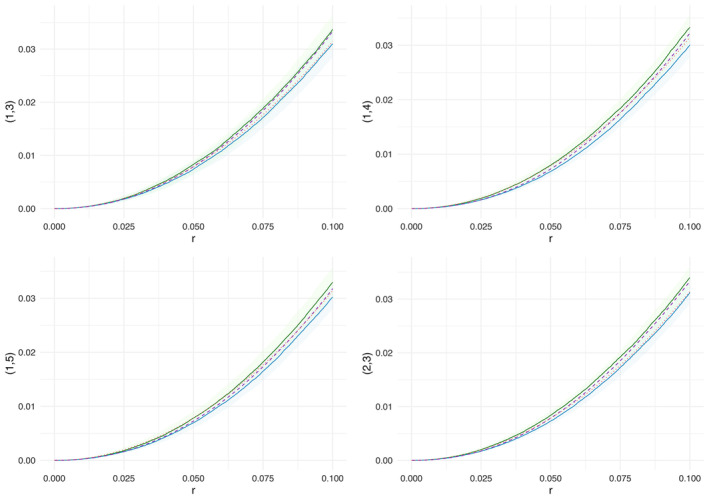
Comparison of empirical and fitted K functions for the five species simulated data. Red dotted line—baseline K function ($\pi r^{2}$); dashed purple line—empirical K function from the data; green line—point‐wise average K function from the MLGCP model; blue line—point‐wise average K function from the SPIGPP model; blue band—simulated envelope from the SPIGPP model; green band—simulated envelope from the MLGCP model. The y‐axis labels indicate the species being compared. For example, in the top‐left graph, the label (1,3), compares the K functions of between‐species interactions between species 1 and species 3.

**FIGURE B.4:**
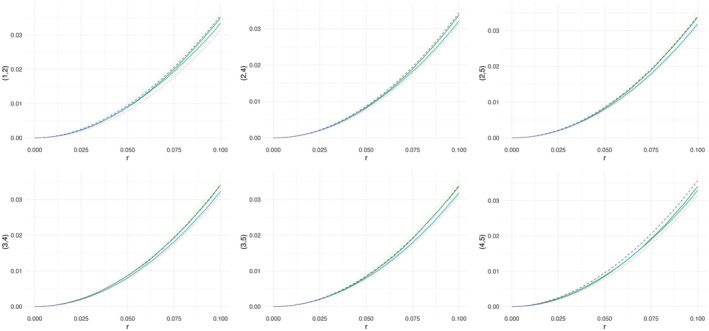
Comparison of empirical and fitted K functions for the five species simulated data. Red dotted line—baseline K function ($\pi r^{2}$); dashed purple line—empirical K function from the data; green line—point‐wise average K function from the MLGCP model; blue line—point‐wise average K function from the SPIGPP model; blue band—simulated envelope from the SPIGPP model; green band—simulated envelope from the MLGCP model. The y‐axis labels indicate the species being compared. For example, in the top‐left graph, the label (1,2), compares the K functions of between‐species interactions between species 1 and species 2.

## Data Availability

R scripts used to generate simulated data and analyses can be found in Github at https://github.com/chathuri‐sam/Modeling‐Spatial‐Dependence‐in‐Multi‐Species‐Point‐Patterns.git.

## Appendix A Simulation Study—Fitting Procedure

In this section, we delved into the simulation and fitting procedures of the comprehensive two‐species simulation study discussed in Section 3. We followed the same format as in Section 3. and explained the fitting procedure in two parts: (1) MLGCP scenarios and (2) SPIGPP scenarios.

### MLGCP Scenarios

All processes under MLGCP scenarios were confined within a unit square window, featuring a single covariate $Z\left(.\right)$ and a background intensity $\rho_{0}\left(.\right)=150\exp\left(0.5V\left(.\right)-\frac{{\left(0.5\right)}^{2}}{2}\right)$. Here, the covariate Z and the background intensity V were zero‐mean unit variance Gaussian random fields with exponential and Gaussian correlation functions with following parameter choices. For all scenarios, $\text{Corr}\left(Z\left(u\right),Z\left(v\right)\right)=\exp\left(\frac{-\|u-v\|}{0.5}\right)$ and $\text{Corr}\left(V\left(u\right),V\left(v\right)\right)$ was set to $\exp\left(-{\left(\frac{\left\|u-v\right\|}{0.8}\right)}^{2}\right)$.

**TABLE A.1:** Initial parameter choices of scenarios when simulating with MLGCP.

	α	σ	ϕ	ξ
MLGCP scenario 1	$\left[\begin{array}{cc} -0.1 & 0.5\\ 0.1 & -0.5 \end{array}\right]$	$\left[\begin{array}{c} 0.5\\ 0.2 \end{array}\right]$	$\left[\begin{array}{c} 0.5\\ 0.5 \end{array}\right]$	0.5
MLGCP scenario 2	$\left[\begin{array}{cc} -0.6 & 0.1\\ 0.6 & -0.1 \end{array}\right]$	$\left[\begin{array}{c} 0.8\\ 0.6 \end{array}\right]$	$\left[\begin{array}{c} 0.5\\ 0.1 \end{array}\right]$	$\left[\begin{array}{c} 0.5\\ 0.1 \end{array}\right]$
MLGCP scenario 3	$\left[\begin{array}{cc} 0.1 & -0.9\\ -0.1 & 0.9 \end{array}\right]$	$\left[\begin{array}{c} 0.5\\ 0.02 \end{array}\right]$	$\left[\begin{array}{c} 0.5\\ 0.08 \end{array}\right]$	0.01
MLGCP scenario 4	$\left[\begin{array}{cc} 1.5 & -0.4\\ -1.5 & 0.4 \end{array}\right]$	$\left[\begin{array}{c} 0.5\\ 0.02 \end{array}\right]$	$\left[\begin{array}{c} 0.5\\ 0.8 \end{array}\right]$	0.05

Different initial distances for short_range distances of 0.5 and 0.8 with exponential models were used. The exponential model meant that the interaction potential was given by $\varphi \left(x\right)=\exp\left(\frac{-\log\left(2\right)x}{R}\right)$ where R was the aforementioned short_range distance. Moreover, the initial values min_dummy = 4000, dummy_factor = 5 and dummy_distribution = ‘stratified’ were used in all the SPIGPP fits for the scenarios. This meant that the dummy points were distributed as a stratified point process where each species with n number of points had max(5*n,4000) dummy points. The fitting_package used was glmnet with a saturation parameter of 4. In the SPIGPP fitting procedure, both the simulated covariate and the background intensity were regarded as covariates. The background intensity served as a covariate due to the absence of analogous settings in SPIGPP compared to MLGCP. Incorporating the background intensity allowed for the comprehensive utilisation of available data without any loss of information during the model fitting process. Other initial parameter choices for α,σ,ϕ and ξ are listed in Table A.1.

### SPIGPP Scenarios

Similar to the MLGCP scenarios, the SPIGPP processes were generated within a unit window, utilising a shared normalised covariate for both species. In SPIGPP models, the parameters $\beta_{0}$ and $\alpha_{p}$ jointly controlled the number of points generated. The expected number of points for each species in all scenarios was set to 100. We employed $10^{5}$ steps in the Metropolis‐Hastings algorithm for all scenarios, with a saturation parameter of 2. Table A.2 lists the initial parameter choices for the simulated SPIGPP scenarios.

**TABLE A.2:** Initial parameter choices of scenarios when simulating with SPIGPP.

	$\beta_{0}$	β	Model	short_range	$\alpha_{p}$
Scenario 1	(4.8, 4.5)	(1.5, 2)	exponential	$\left[\begin{array}{cc} 0.05 & 0.05\\ 0.05 & 0.05 \end{array}\right]$	$\left[\begin{array}{cc} 0.02 & 0.05\\ 0.05 & 0.04 \end{array}\right]$
Scenario 2	(3.2, 2.2)	(1.5, 2)	square_exponential	$\left[\begin{array}{cc} 0.05 & 0.05\\ 0.05 & 0.05 \end{array}\right]$	$\left[\begin{array}{cc} 0.4 & 0.6\\ 0.6 & 0.9 \end{array}\right]$
Scenario 3	(4.8, 4.5)	(1.5, 2)	exponential	$\left[\begin{array}{cc} 0.05 & 0.05\\ 0.05 & 0.05 \end{array}\right]$	$\left[\begin{array}{cc} 0.2 & -0.5\\ -0.5 & 0.4 \end{array}\right]$
Scenario 4	(5, 4.1)	(−1, 1)	square_bump	$\left[\begin{array}{cc} 0.05 & 0.05\\ 0.05 & 0.05 \end{array}\right]$	$\left[\begin{array}{cc} -0.4 & -0.1\\ -0.1 & 0.3 \end{array}\right]$
Scenario 5	$\left(3.6,3.5\right)$	(1.5, 2)	exponential	$\left[\begin{array}{cc} 0.05 & 0.05\\ 0.05 & 0.05 \end{array}\right]$	$\left[\begin{array}{cc} 0.9 & -0.5\\ -0.5 & 0.9 \end{array}\right]$

**TABLE A.3:** Initial values chosen to fit the generated SPIGPP processes with MLGCP models.

Scenarios	ξ	σ	ϕ	Latent
Scenarios 1/2	(0.05, 0.01)	(1, 0.01)	(0.05, 0.01)	1
Scenarios 2	(0.03, 0.01)	(0.8, 0.1)	(0.03, 0.01)	1
Scenario 5	(0.03, 0.01)	(0.8, 0.1)	(0.03, 0.01)	2

When fitting the MLGCP models to the SPIGPP scenarios outlined in Table A.2, we employed the initial values specified in Table A.3 without any regularisation (λ=0). We also estimated the background intensity $\left(\rho_{0}\right)$ from data using the approach stated in Hessellund et al. (2022a). Then, we proceeded to compare the fitted models as discussed in Section 3.1.

## Appendix B Evaluating SPIGPP Model Performance on Five‐Variate LGCP

This segment of our simulation followed the study conducted in Waagepetersen et al. (2016), later revisited by Choiruddin et al. (2020); Jalilian et al. (2020), and extensively explored in Hessellund et al. (2022a). Our objective in this phase was to utilize these simulations to gain a comprehensive understanding of the joint performance of SPIGPP models when used with mis‐specified models. Additionally, we opted to compare the performance of the SPIGPP fit with the second‐order conditional composite likelihood, as outlined in Hessellund et al. (2022a).

We generated a five‐variant point process, denoted as $X={\left(X_{1},X_{2},X_{3},X_{4},X_{5}\right)}^{T}$, over the spatial domain $W={\left[0,1\right]}^{2}$. This simulation was based on two distinct settings, where X was modelled as a multivariate LGCP. We also generated a single covariate $Z\left(.\right)$ and a background intensity $\rho_{0}\left(.\right)=400\exp\left(0.5V\left(.\right)-\frac{0.5^{2}}{2}\right)$, where Z and V represented zero‐mean unit‐variance Gaussian random fields with exponential and Gaussian correlation functions, respectively, as employed in Hessellund et al. (2022a). The realisations of Z and $\rho_{0}$ are illustrated in Figure B.1, and these realisations remain constant throughout the entire simulation study.

Table B.1 provided the values used for the intensity function regression parameters γ, along with the standard deviation σ and correlation scale parameters ϕ for the type‐specific latent fields Z and V. We set q=2, and $\xi_{1}=0.02$ and $\xi_{2}=0.03,$ with $\alpha ={\left[\begin{array}{ccccc} 0.5 & 0.5 & -1 & 0 & 0\\ -1 & 0 & 0 & 0.5 & 0.5 \end{array}\right]}^{T}$. In this case, a positive spatial dependence existed between $X_{1}$ and $X_{2}$ and between $X_{4}$ and $X_{5}$, while negative spatial dependence was observed between $X_{3}$ and $\left(X_{1},X_{2}\right)$ and between $X_{1}$ and $\left(X_{4},X_{5}\right)$.

**TABLE B.1:** Simulation settings for X in each setup q=0,2 (excluding α and ξ).

X	$\gamma_{0}$	$\gamma_{1}$	σ	ϕ
$X_{1}$	0.1	−0.1	0.71	0.02
$X_{2}$	0.2	−0.2	0.71	0.02
$X_{3}$	0.3	0	0.71	0.03
$X_{4}$	0.4	0.1	0.71	0.03
$X_{5}$	0.5	0.2	0.71	0.04

Subsequently, we applied SPIGPP models to the simulated samples from MLGCP, utilizing a Rectangle_window(c(0,1),c(0,1)) with initial values for the model set to dummy_factor = 5, min_dummy = 1000, dummy_distribution = “stratified”, and saturation = 5. We also used a fitting_package of glmnet and two potentials for model and short_range interaction distances as given in Table B.2. As detailed previously, our focus was on using the K functions to assess the model performance. Additionally, we evaluated the performance of each selected model using the mean integrated squared error (MISE) computed based on the K functions.

**TABLE B.2:** Initial potentials chosen for SPIGPP model parameters of five species MLGCP simulation.

	Model	short_range
Potential 1	“exponential”	matrix(0.05, nrow=5,ncol=5)
Potential 2	“exponential”	matrix(0.2, nrow=5,ncol=5)

The empirical and fitted K functions from MLGCP and SPIGPP models for the within‐species associations with respective confidence bands were presented in Figure B.2. Out of the five species, the K function of the MLGCP fit (green) and the SPIGPP fit (blue) closely followed that of the empirical K function in dashed purple in species 1, 2, and 5. Both MLGCP and SPIGPP fits showed similar deviations from the empirical K functions for species 3 and 4, which was interesting as it was expected for the MLGCP to perform better since the scenario was simulated from MLGCP.

Similar observations applied to the between‐species clustering depicted by the cross K functions in Figure B.4. Empirically, these inter‐species clustered pattern were relatively smaller compared to the intra‐species clustering discussed earlier. The blue solid lines in the figure illustrated that the SPIGPP model adequately captured most of the between‐species clustering, denoted as $\left(1,2\right),\left(2,5\right),\left(3,4\right),\left(3,5\right),\left(4,5\right)$, despite being a mis‐specified model, while MLGCP fits them better. Notably, between‐species clustering $\left(2,4\right)$ stood out as slightly larger than the others and SPIGPP failed to capture the magnitude of the clustered pattern accurately, similarly for MLGCP fitting algorithm proposed by (Hessellund et al. 2022b).

The interactions between species $\left(1,3\right)$, $\left(1,4\right)$, $\left(1,5\right)$, and $\left(2,3\right)$ exhibited empirical K functions showcasing regular pattern initially, transitioning into attractions around r=0.1. In Figure B.3, we demonstrated the close alignment of the SPIGPP model with these empirical dynamics. The blue solid line representing the SPIGPP fit closely overlaid the empirical (purple) K functions during the regular phase at the beginning, as can be seen in Figure B.3, while MLGCP only captured (over‐estimated) the clustering between them and was unable to identify the regular associations at the beginning.

Based on our investigation into this five‐variate LGCP simulation, we observed that the SPIGPP model accurately identified clustered and repulsive associations when there were no transitions from clustering to regularity or vice versa within a single species. However, when there were fluctuations with distance between clustered and/or repulsive associations, the SPIGPP model effectively captured the interaction in short ranges but struggled to accurately represent the transitions in the interaction, while MLGCP tended to capture the clustering at the longer distances. This limitation in SPIGPP may have arisen from the disparities in the underlying Cox and Gibbs processes between MLGCP and SPIGPP models. We may have been able to get a better fit by using medium_range and/or long_range in SPIGPP.

## Appendix C Case Study—Fitting Procedure

In this section, we delved into the details of the fitting procedure used in the case study discussed in Section 4.

In the MLGCP fitting process, we set q=2 and a regularisation parameter λ=2.5. For the second‐order composite likelihood, a distance parameter of R=200 meters was used with the covariate for the water level. Both models assumed a rectangle window of $\left(0,200\right)\times \left(0,50\right)$ around the area where the points were distributed.

When fitting SPIGPP models, the covariate for the water level and the estimated background intensity $\rho_{0}$ were used as covariates to ensure a fair comparison with the MLGCP models fitted. We also used the parameter choices provided in Table C.1.

**TABLE C.1:** SPIGPP models' initial value choices.

	Model
short_range	matrix(5, 5, 5)
Model	square_exponential
dummy_factor	1
min_dummy	5000
dummy_distribution	stratified
fitting_package	glm
Saturation	2

To estimate $\rho_{0}$, we employed the semi‐parametric kernel estimator outlined in Section 5 of the supplementary documents in Hessellund et al. (2022a). This involved sub‐setting the dataset for each tree type and fitting regression models, incorporating an intercept and the covariate (water level), utilising the function ppm in spatstat package in R statistical software. Then the intensity at each tree location was predicted using the intensity function. Following this, the density for each tree was computed, incorporating the intensity as weights in the density function. In this step, we selected the bandwidth based on the criterion inspired by Cronie and van Lieshout (2018), implemented through the bw.CvL. Finally, the density across all tree types were averaged to obtain the estimated background intensity $\rho_{0}$ and is shown in Figure C.1.

**TABLE C.2:** MLGCP parameter estimates for each tree type for $\left(q,\lambda \right)=\left(2,2.5\right)$.

Tree type	$\hat{\alpha_{.2}}$	$\hat{\sigma }$	$\hat{\phi }$
Carolina ash	0.565	2.236	0.668
Swamp tupelo	−0.645	0.660	3.891
Water tupelo	−0.356	1.401	2.295
other	0.230	2.0334	1.999
Bald cypress	0.205	1.091	5.659

The below, $\alpha_{p}$ matrix supported the result given in Figure 5 in Section 4 . In the estimated $\alpha_{p}$ matrix, we observed repulsive associations between all species. However, when computing the K functions, we also found a few clustering. These clustering occurred between the tree species Carolina Ash and Bald Cypress and between other species and Bald Cypress. These K functions showed clustered patterns due to the fact that they were not significant at the 0.05 level of significance when estimating the short_range $\alpha_{p}$ matrix.

Figure C.2 displayed the log‐papangelou conditional intensities (Flint et al. 2022) of the tree species in the fitted SPIGPP model (which was explained in Section 4).

For the comparison of the two methods, we computed the K functions. To derive these K functions, we simulated 100 fitted MLGCPs using the estimated parameters such as α, σ, ξ, ϕ, estimated *β*s, and $\log\left(\hat{\rho_{0}}\right)$. These simulated fitted MLGCP samples were then used to compute the MLGCP K functions. Similarly, utilising the fitted parameters from each model, we simulated 100 fitted SPIGPP samples to generate the fitted approximate K functions for the SPIGPP models. We then compared the K functions of the fitted models (MLGCP and SPIGPP) with the empirical K function computed from the data.

## References

[ece371066-bib-0001] Baddeley, A. , J.‐F. Coeurjolly , E. Rubak , and R. Waagepetersen . 2014a. “Logistic Regression for Spatial Gibbs Point Processes.” Biometrika 101, no. 2: 377–392.

[ece371066-bib-0002] Baddeley, A. , A. Jammalamadaka , and G. Nair . 2014b. “Multitype Point Process Analysis of Spines on the Dendrite Network of a Neuron.” Journal of the Royal Statistical Society, Series C 63: 673–694.

[ece371066-bib-0003] Baddeley, A. , E. Rubak , and R. Turner . 2016. Spatial Point Patterns—Methodology and Applications With R. CRC Press.

[ece371066-bib-0004] Choiruddin, A. , F. Cuevas‐Pacheco , J.‐F. Coeurjolly , and R. Waagepetersen . 2020. “Regularized Estimation for Highly Multivariate Log Gaussian Cox Processes.” Statistics and Computing 71, no. 5: 1721–1752.

[ece371066-bib-0005] Cronie, O. , and M. van Lieshout . 2016. “Summary Statistics for Inhomogeneous Marked Point Processes.” Annals of the Institute of Statistical Mathematics 68: 905–928.

[ece371066-bib-0006] Cronie, O. , and M. van Lieshout . 2018. “A Non‐Model‐Based Approach to Bandwidth Selection for Kernel Estimators of Spatial Intensity Functions.” Biometrika 105: 455–462.

[ece371066-bib-0007] Daley, D. , and D. Vere‐Jones . 2003. An Introduction to the Theory of Point Processes. Vol. 1. Springer‐Verlag.

[ece371066-bib-0008] Dixon, P. 2002. “Nearest‐Neighbor Contingency Table Analysis of Spatial Segregation for Several Species.” Ecoscience 9: 142–151.

[ece371066-bib-0009] Flint, I. , N. Golding , P. Vesk , Y. Wang , and A. Xia . 2022. “The Saturated Pairwise Interaction Gibbs Point Process as a Joint Species Distribution Model.” Journal of the Royal Statistical Society: Series C: Applied Statistics 71: 1721–1752.

[ece371066-bib-0010] Good, B. , and S. Whipple . 1982. “Tree Spatial Patterns: South Carolina Bottomland and Swamp Forests.” Bulletin of the Torrey Botanical Club 109: 529–536.

[ece371066-bib-0011] Hessellund, K. B. , G. Xu , Y. Guan , and R. Waagepetersen . 2022a. “Second‐Order Semi‐Parametric Inference for Multivariate Log Gaussian Cox Processes.” Journal of the Royal Statistical Society: Series C: Applied Statistics 71, no. 1: 244–268.

[ece371066-bib-0012] Hessellund, K. B. , G. Xu , Y. Guan , and R. Waagepetersen . 2022b. “Semiparametric Multinomial Logistic Regression for Multivariate Point Pattern Data.” Journal of the American Statistical Association 117, no. 539: 1500–1515.

[ece371066-bib-0013] Jalilian, A. , A. Safari , and H. Sohrabi . 2020. “Modeling Spatial Patterns and Species Associations in a Hyrcanian Forest Using a Multivariate Log‐Gaussian Cox Process.” Journal of Statistical Modelling: Theory and Applications 1, no. 2: 59–76.

[ece371066-bib-0014] Jones, R. H. , R. R. Sharitz , S. M. James , and P. Dixon . 1994. “Tree Population Dynamics in Seven South Carolina Mixed‐Species Forests.” Bulletin of the Torrey Botanical Club 121: 360–368.

[ece371066-bib-0015] Møller, J. , and R. P. Waagepetersen . 2003. Statistical Inference and Simulation for Spatial Point Processes. Chapman and Hall.

[ece371066-bib-0018] R Core Team . 2024. R: A Language and Environment for Statistical Computing. R Foundation for Statistical Computing.

[ece371066-bib-0016] Rajala, T. , D. Murrell , and S. Olhede . 2018. “Detecting Multivariate Interactions in Spatial Point Patterns With Gibbs Models and Variable Selection.” Journal of the Royal Statistical Society Series C, Royal Statistical Society 67: 1237–1273.

[ece371066-bib-0017] Waagepetersen, R. , Y. Guan , A. Jalilian , and J. Mateu . 2016. “Analysis of Multispecies Point Patterns by Using Multivariate Log‐Gaussian Cox Processes.” Journal of the Royal Statistical Society: Series C: Applied Statistics 65: 77–96.

